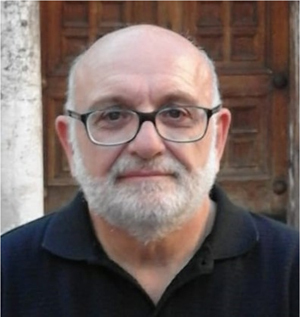# In memoriam Dr. Josep Maria Queraltó, esteemed member of the SEQC^ML^ for 35 years

**DOI:** 10.1515/almed-2020-0076

**Published:** 2020-09-03

**Authors:** Board of Directors of the Spanish Society of Laboratory Medicine

The Spanish Society for Laboratory Medicine (SEQC^ML^) regrets having to report the death of one of its most illustrious members, Dr. Josep Maria Queraltó, 67 years old, who left us suddenly, on Friday, March 27, at his home in Sant Andreu de Llavaneres (Barcelona).

Dr. Queraltó had been a very involved member of the Spanish Society of Laboratory Medicine for 35 years. His activities at the national level included his position as secretary of the Board of Directors of the Society from 1990 to 1996, the presidency of the former Reference Values Commission and the Education Committee during the 80s and 90s, and the presidency of the Drugs Monitoring and Clinical Toxicology Commission during two periods (2004–2005 and 2013–2018).

In addition, he was a member of the Commission on the Diagnostic Value of Biochemical magnitudes (1985–1989) and of the Scientific Committee of the Society (1985–1992), as well as of the Editorial Committee of the Journal Advances in Laboratory Medicine and the Board of Directors; he was still holding the last position at the time of his death.

Likewise, his work representing SEQC^ML^ in international organizations has been of great importance, as he held highly significant positions, such as the presidency of the board of the European Federation of European Societies of Clinical Chemistry. He was also a member of the EC4 European Registry Committee from 2000 to the present.

His perseverance in the many international tasks in which he participated and his international contacts, which he cultivated and maintained exquisitely, helped the SEQC^ML^ to be present at numerous international events. Among these it is worth noting, due to its great impact, the organization of the congresses EuroMedLab Barcelona 2003, of which he was president of the Scientific Committee, and EuroMedLab Barcelona 2019, of which he was a member of the Organizing Committee. The success of both European Congresses gave the SEQC^ML^ an enormous boost and visibility at an international level.

In the days since his death, the Society has received dozens of emails and written statements from various countries around the world, expressing sorrow and dismay at the loss of such a beloved and valued person. Within his great human and professional worth, all those who had the opportunity to interact with him highlighted his patience and his great vision for the future in the field of Laboratory Medicine training.

**Figure:**
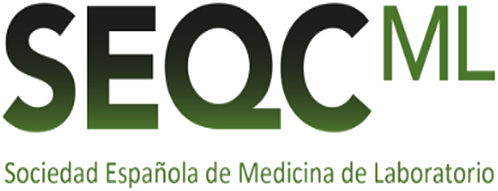

**Figure:**